# Metagenomic Insights into the Metabolic and Ecological Functions of Abundant Deep-Sea Hydrothermal Vent DPANN Archaea

**DOI:** 10.1128/AEM.03009-20

**Published:** 2021-04-13

**Authors:** Ruining Cai, Jing Zhang, Rui Liu, Chaomin Sun

**Affiliations:** a CAS Key Laboratory of Experimental Marine Biology & Center of Deep Sea Research, Institute of Oceanology, Chinese Academy of Sciences, Qingdao, China; b Laboratory for Marine Biology and Biotechnology, Pilot National Laboratory for Marine Science and Technology, Qingdao, China; c College of Earth Science, University of Chinese Academy of Sciences, Beijing, China; d Center of Ocean Mega-Science, Chinese Academy of Sciences, Qingdao, China; Kyoto University

**Keywords:** DPANN, hydrothermal vents, metabolism, ecology significance

## Abstract

Due to their unique metabolism and important ecological roles, deep-sea hydrothermal archaea have attracted great scientific interest. Among these archaea, DPANN superphylum archaea are widely distributed in hydrothermal vent environments. However, DPANN metabolism and ecology remain largely unknown. In this study, we assembled 20 DPANN genomes among 43 reconstructed genomes obtained from deep-sea hydrothermal vent sediments. Phylogenetic analysis suggests 6 phyla, comprised of *Aenigmarchaeota, Diapherotrites, Nanoarchaeota, Pacearchaeota, Woesearchaeota,* and a new candidate phylum we have designated *Kexuearchaeota*. These are included in the 20 DPANN archaeal members, indicating their broad diversity in this special environment. Analyses of their metabolism reveal deficiencies due to their reduced genome size, including gluconeogenesis and *de novo* nucleotide and amino acid biosynthesis. However, DPANN archaea possess alternate strategies to address these deficiencies. DPANN archaea also have the potential to assimilate nitrogen and sulfur compounds, indicating an important ecological role in the hydrothermal vent system.

**IMPORTANCE** DPANN archaea show high distribution in the hydrothermal system, although they display small genome size and some incomplete biological processes. Exploring their metabolism is helpful to understand how such small forms of life adapt to this unique environment and what ecological roles they play. In this study, we obtained 20 high-quality metagenome-assembled genomes (MAGs) corresponding to 6 phyla of the DPANN group (*Aenigmarchaeota, Diapherotrites, Nanoarchaeota, Pacearchaeota, Woesearchaeota,* and a new candidate phylum designated *Kexuearchaeota*). Further metagenomic analyses provided insights on the metabolism and ecological functions of DPANN archaea to adapt to deep-sea hydrothermal environments. Our study contributes to a deeper understanding of their special lifestyles and should provide clues to cultivate this important archaeal group in the future.

## Abstract (precis)

DPANN archaea show high distribution in the hydrothermal system, although they display small genome size and some incomplete biological processes. Exploring their metabolism is helpful to understand how such small forms of life adapt to this unique environment and what ecological roles they play.

## INTRODUCTION

Archaea are important microorganisms and play key roles in energy flows and biogeochemical cycles (1–3). Previously, all archaea were identified by cultivation methods and assigned to two clades: *Crenarchaeota* and *Euryarchaeota* (4). With the development of novel sequencing techniques, such as metagenome analysis, the archaeal tree has dramatically expanded (5). To date, at least four supergroups have been described in the archaeal domain: *Euryarchaeota*, TACK, Asgard, and DPANN (4). Furthermore, newly discovered archaeal genes and functions have provided a better understanding of the evolution, lifestyle, and ecological significance of these archaea (6).

Within the archaeal domain, DPANN are a superphylum first proposed in 2013 (7). DPANN consist of at least 10 phylum-level lineages, including *Altiarchaeota, Diapherotrites, Aenigmarchaeota, Pacearchaeota, Woesearchaeota, Micrarchaeota, Parvarchaeota, Nanohaloarchaeota, Nanoarchaeota,* and *Huberarchaeota*. DPANN lineages form a candidate superphylum and have attracted great scientific interest (5, 8–10). Recently, the lifestyles and ecology of DPANN groups collected from different biotopes were analyzed and summarized using culture-independent methods (7, 8, 11, 12). The primary biosynthetic pathways of DPANN groups include amino acid biosynthesis, nucleotide biosynthesis, and lipid biosynthesis, and vitamin biosynthesis pathways were absent (10). Furthermore, most DPANN groups lack complete tricarboxylic acid (TCA) cycles and pentose phosphate pathways (8). However, it has been suggested that DPANN groups utilize a putative ferredoxin-dependent complex 1-like oxidoreductase for generating a proton-motive force and driving ATP synthesis (11). Similarly, many DPANN genomes contain genes encoding enzymes involved in generating fermentation products, such as lactate, formate, ethanol, and acetate, strongly suggesting they have evolved to use substrate-level phosphorylation as a mode of energy (8, 12).

Due to their small genomes and limited metabolism, DPANN were inferred to be dependent on symbiotic interactions with other organisms and to even be parasitic (11). Several reports recognized that *Nanoarchaeota* and *Parvarchaeota* required contacting similarly or larger-sized hosts to proliferate or parasitize (13–16). *Huberarchaeota* were also described as a possible epibiotic symbiont of “*Candidatus* Altiarchaeum” spp. (17). *Woesearchaeota* were revealed to possess a potential syntrophic relationship with methanogens and were thought to impact methanogenesis in inland ecosystems (7). Lastly, *Woesearchaeota* were also found to be ubiquitous in petroleum reservoirs, where they contributed to ecosystem diversity and biogeochemical carbon cycles (18).

Previous studies have been conducted on DPANN groups derived from different biotopes, such as groundwater (19), surface water (20), and marine sediments (21). However, DPANN groups living in deep-sea hydrothermal vent sediments have not been investigated, despite their high abundance within this ecosystem (22, 23). Microbes living in deep-sea hydrothermal vent sediments drive nutrient cycling within the ecosystem (24, 25). Furthermore, deep-sea hydrothermal vent microflora have specific physiological characteristics to adapt to their unique habitats (26). Most importantly, deep-sea vent microbes are thought to contain crucial insights into understanding specialized life processes and how organisms survive in special environments. Since DPANN are abundant in the archaeal domain of hydrothermal vent systems, it is critical to better understand their lifestyles, metabolism, and ecological functions as part of global biogeochemical cycles (23).

Here, we found DPANN were abundant in Western Pacific deep-sea hydrothermal vent system. Using genomic analyses, we characterized DPANN lifestyles and metabolism within this specialized environment. Lastly, we found that DPANN possessed multiple capacities for metabolizing carbon, nitrogen, and sulfur compounds, and that, through these functions, they likely played a critical role within hydrothermal vent systems, despite their minimal genomes and limited capacities to synthesize amino acids and nucleotides.

## RESULTS AND DISCUSSION

### DPANN are highly abundant and broadly dispersed in hydrothermal systems.

**FIG 1:**
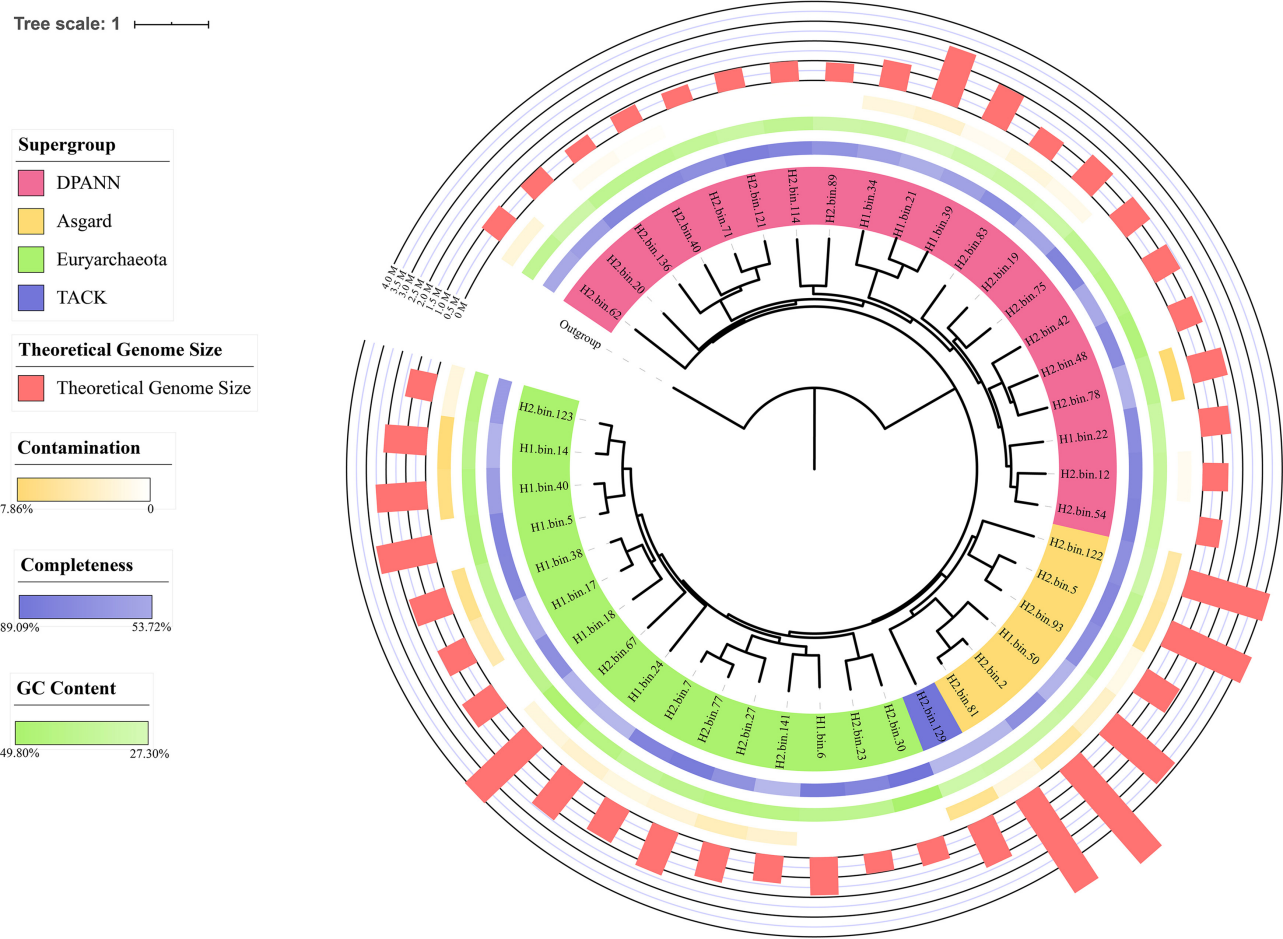
Phylogeny of 43 assembled genomes from hydrothermal vent sediments. The maximum likelihood tree based on 37 single-copy protein-coding genes is shown. Different supergroups are colored in red (DPANN), yellow (Asgard), green (Euryarchaeota), and blue (TACK) within the corresponding leaves in the tree. The uncolored leaf represents an outgroup. Outer colored circles indicate the completeness (blue), GC content (green), contamination (yellow), and theoretical genome size (red) of assembled genomes. Referenced information is available in Data Set S1.

To explore the composition and specificity of archaeal communities inhabiting deep-sea hydrothermal vent sediments, we first chose two sampling sites in the Okinawa Trough of the Western Pacific (see Fig. S1 and Table S1 in the supplemental material). The predominant characteristics in both these deep-sea hydrothermal areas are a faint glow from black smokers and a large number of shrimp (Fig. S2). We collected and whole-genome sequenced two sediment samples from these locations. We generated 43 metagenome-assembled genomes (MAGs) from these samples. All assembled genomes reached >50% completeness, with 26 genomes reaching >70% completeness and <10% contamination (Data Set S1). We classified these MAGs by generating a phylogenetic tree using the sequences of 37 single-copy protein-coding marker genes (Table S2). Overall, 43 MAGs group into 19 unique lineages based on phylogenetic distance analyses (branch length distance, <0.6) (Fig. S3 and Data Set S2). As such, we obtained 20 DPANN MAGs (Fig. 1 and Fig. S3) from hydrothermal vent sediments, suggesting DPANN are highly abundant in this Western Pacific deep-sea hydrothermal vent system.

**FIG 2:**
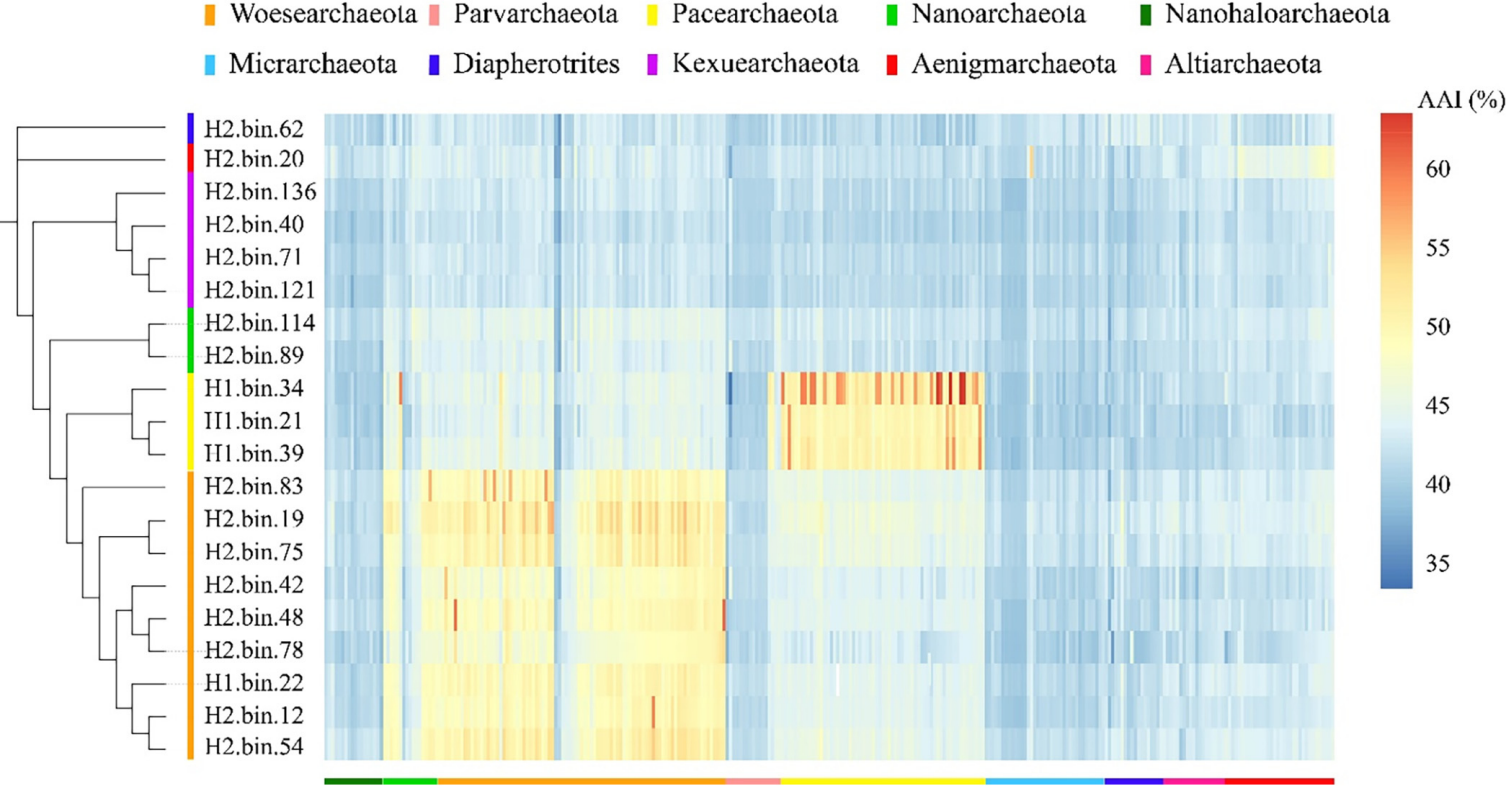
Genome-identity correlation matrix of assembled genomes compared with reference DPANN genomes. Referenced genomes containing all DPANN genome sequences were downloaded from NCBI. Amino acid identity correlation matrix was calculated by CompareM. Data are available in Data Set S4.

To further characterize the phylum categories of the DPANN group, which we have named DPANN-HV, we performed phylogenetic analyses using the same methods described above (Fig. S4, Data Set S3). In total, the 20 MAGs we obtained represent 6 distinct phyla, comprising *Aenigmarchaeota* (*n* = 1), *Diapherotrites* (*n* = 1), *Nanoarchaeota* (*n* = 2), *Pacearchaeota* (*n* = 3), *Woesearchaeota* (*n* = 9), and a new candidate phylum, designated *Kexuearchaeota* (*n* = 4). *Kexuearchaeota* formed a distinct clade in the phylogenetic tree (Fig. S4). This further supports the designation of *Kexuearchaeota* as a new candidate phylum generated by directly comparing the average amino acid identity (AAI) against all public DPANN MAGs in the NCBI RefSeq database (Fig. 2, Data Set S4). The AAI value of *Kexuearchaeota* is less than 46.11%, which is lower than the threshold considered for separate phyla (27) (Fig. S5, Data Set S4). Overall, these pieces of evidence strongly indicate *Kexuearchaeota* can be described as a new phylum in the DPANN group, suggesting specific DPANN adaptations exist for living in such an extreme environment.

### DPANN-HV lack gluconeogenesis pathway but possess alternative carbohydrate metabolism.

**FIG 3:**
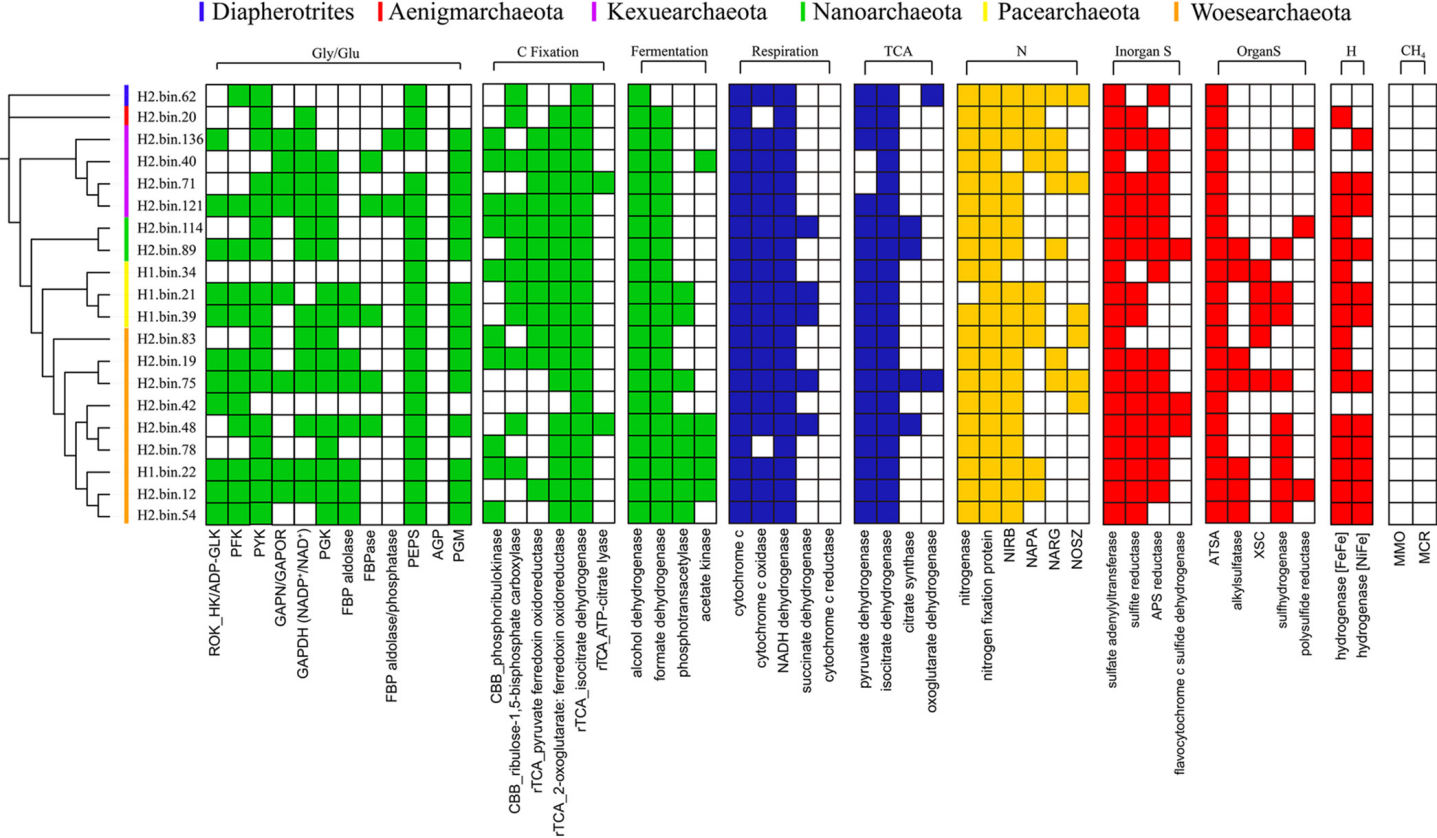
Core metabolic genes detected in DPANN-HV assembled genomes. Core metabolic genes for carbon metabolism, respiration, nitrogen metabolism, and sulfur metabolism are shown. Gene predictions are based on sequence alignments with KEGG, NR, and UniProt databases. Solid colors indicate the gene exists in the assembled genome. White indicates the gene is absent from this assembled genome. ROK_HK/ADP-GLK, ROK hexokinase/ADP-dependent glucokinases; PFK, phosphofructokinases; PYK, pyruvate kinase; GAPN/GAPOR, nonphosphorylating glyceraldehyde-3-phosphate dehydrogenase/ferredoxin (Fd)-dependent glyceraldehyde-3-phosphate oxidoreductase; GAPDH (NADP^+^/NAD^+^), glyceraldehyde-3-phosphate dehydrogenase (NADP^+^/NAD^+^); PGK, phosphoglycerate kinase; FBP aldolase, fructose-1,6-bisphosphate aldolase; FBP aldolase, fructose-1,6-bisphosphate aldolase; FBPase, fructose-1,6-bisphosphatase; FBP aldolase/phosphatase, fructose-1,6-bisphosphate aldolase/phosphatase (bifunctional); PEPS, phosphoenolpyruvate synthase; AGP, glucose-1-phosphatase; PGM, phosphoglucomutase; CBB_Phosphoribulokinase, phosphoribulokinase; CBB_ribulose-1,5-bisphosphate carboxylase, ribulose-1,5-bisphosphate carboxylase; rTCA_pyruvate, ferredoxin oxidoreductase; rTCA_2-oxoglutarate, ferredoxin oxidoreductase; 2-oxoglutarate, ferredoxin oxidoreductase; rTCA_isocitrate dehydrogenase, isocitrate dehydrogenase; rTCA_ATP-citrate lyase, ATP-citrate lyase; NIRB, nitrite reductase encoded by *nirB*; NAPA, nitrate reductase encoded by *napA*; NOSZ, nitrous-oxide reductase encoded by *nosZ*; NARG, respiratory nitrate reductase encoded by *narG*; APS reductase, adenosine-5′-phosphosulfate reductase; ATSA, arylsulfatase encoded by *atsA*; XSC, sulfoacetaldehyde acetyltransferase encoded by *xsc*; MMO, methane monooxygenase; MCR, methyl coenzyme M reductase.

Assembled genomes from DPANN-HV were smaller than other archaeal genomes (Fig. S6, Data Set S3), which is consistent with previous reports (11). To determine the specific metabolism of these small-genome archaea, we first tested their ability to metabolize carbohydrates, as glycolysis plays an essential energetic role in many organisms through the breakdown of glucose. Genes encoding glycolysis enzymes were searched for in the DPANN-HV MAGs (28). In the DPANN-HV MAGs, we found core genes of the modified Embden-Meyerhof-Parnas (EMP) pathway encoding nonphosphorylating glyceraldehyde-3-phosphate dehydrogenase (GAPN) and ferredoxin (Fd)-dependent glyceraldehyde-3-phosphate oxidoreductase (GAPOR) (Fig. 3, Fig. S7, Data Set S5). In addition to these specific genes, we also found other genes of the modified EMP pathway. These results indicate DPANN-HV prefer to use modified EMP pathways for glycolysis. In addition, genes encoding phosphoglycerate kinase (PGK) and pyruvate kinase (PYK), which are responsible for substrate-level phosphorylation, were identified in 75% of DPANN-HV MAGs. This suggests DPANN-HV utilizes glycolysis for energy conservation. Counter to glycolysis, gluconeogenesis, which synthesizes the six-carbon sugar and other important metabolic intermediates, like fructose 6-phosphate, is crucial for most living organisms. Unexpectedly, only 6 assembled genomes have fructose-1,6-bisphosphatase (FBPase) genes and also lack reversible PP_i_-dependent phosphofructokinase genes (Fig. 3, Fig. S8, Table S4). This likely means that most DPANN-HV have incomplete gluconeogenesis pathways and do not convert fructose 1,6-bisphosphate to fructose 6-phosphate (Fig. 3) (28, 29).

**FIG 4:**
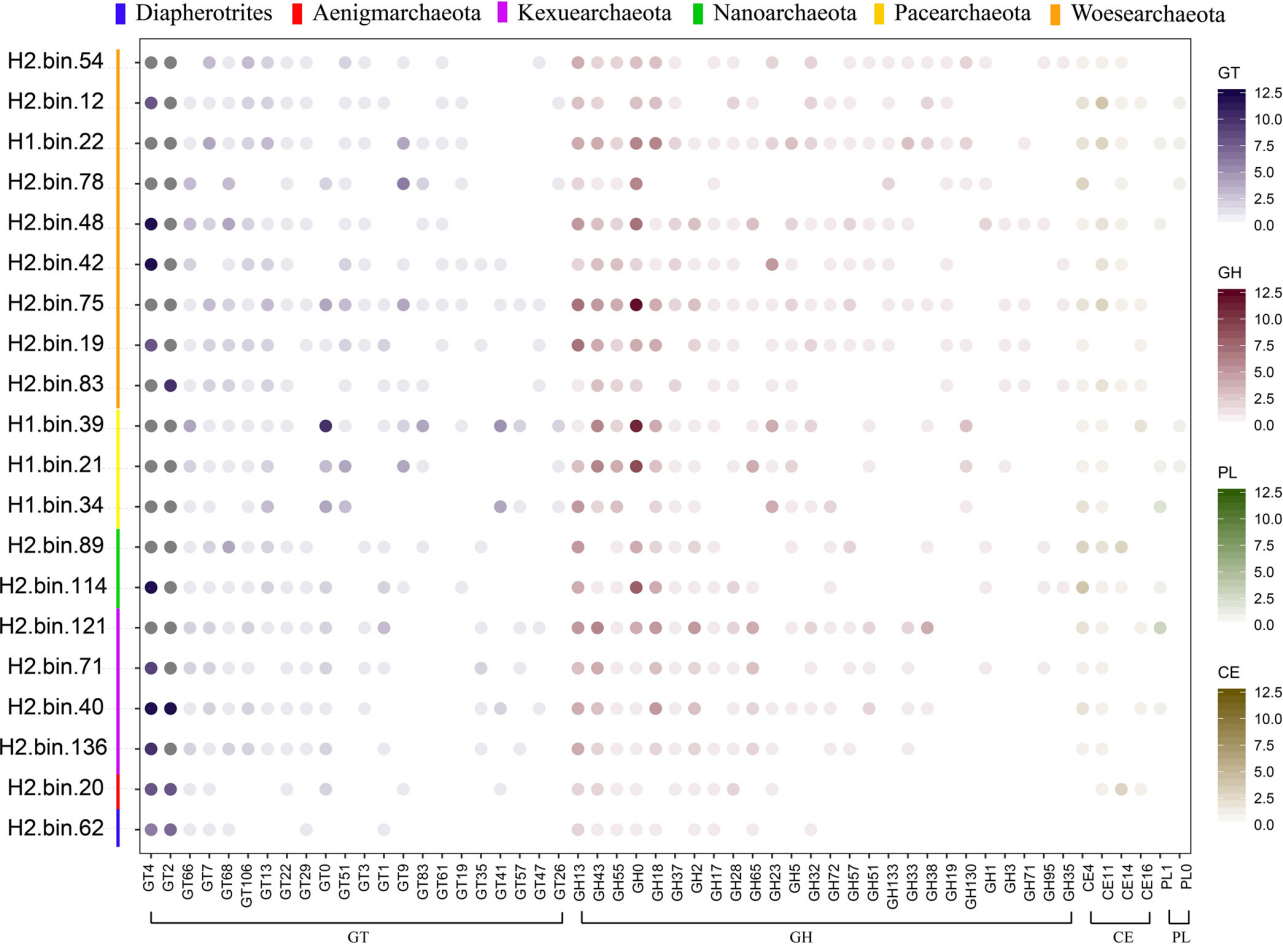
Relative abundance of carbohydrate-active enzyme (CAZymes) genes in DPANN-HV assembled genomes. The number of carbohydrate esterase (CE), glycoside hydrolase (GH), glycosyltransferase (GT), and polysaccharide lyase (PL) genes in each DPANN-HV genome. The numbers of genes belonging to different CAZyme families per genome are represented by colors in circles. Gene distribution, classification, and functions are reported in Data Set S6.

Glucose is an important substrate for primary metabolism in many microorganisms, and gluconeogenesis is an important pathway to generate glucose from certain noncarbohydrate carbon substrates. Given their lack of gluconeogenesis, we next determined how DPANN-HV obtain sugar for metabolism. Accordingly, we investigated the abilities of DPANN-HV to degrade and metabolize complex carbohydrates and peptides. To assess the DPANN-HV degradation of complex sugars, we used the CAZy database to search for carbohydrate-active enzymes (CAZymes) in DPANN-HV MAGs. The results showed that there were roughly 70 CAZymes per MAG (Fig. 4, Data Set S6). Among these CAZymes, glycoside hydrolases and carbohydrate esterases far outnumbered polysaccharide lyases. We identified only 27 polysaccharide lyases, which are members of pectin-degraded PL1 and unknown PL0, suggesting DPANN-HV have limited polysaccharide decomposition functions in reactions with polysaccharide lyases. It is worth noting that approximately 12% of CAZymes are potentially secreted (Fig. S9, Data Set S6), indicating that complex substrates could be broken down outside the cell and taken up later. These secreted enzymes, including members of GH43, GH18, and others (Fig. S10), facilitate DPANN-HV in degrading complex carbohydrates present in their surroundings, which can then be utilized for cell growth. Additionally, genes encoding secreted peptidases are distributed throughout DPANN-HV MAGs (Fig. S11 and S12, Data Set S6), suggesting that these archaea decompose proteins in the environment. Among these peptidases, collagenases can break down collagen from the environment. Given the abundance of giant tube worms in hydrothermal vents (30), this system may be rich in collagen and supply a sufficient protein source for DPANN-HV.

In addition to carbohydrates and peptides, DPANN-HV may degrade aromatic hydrocarbons, as we identified genes encoding phenylphosphate synthase, the key enzyme responsible for transformation of phenol to phenylphosphate (31), in DPANN-HV MAGs (Fig. S13). As the predominant pathway described previously (32–34), phenylphosphate is converted to fumarate by phenylphosphate carboxylase and other enzymes in the tricarboxylic acid (TCA) cycle (34). However, phenylphosphate carboxylase was not detected in DPANN-HV MAGs. Moreover, given the lack of citrate synthase, oxoglutarate dehydrogenase, and other TCA cycle enzymes, DPANN-HV lack a complete TCA cycle (Fig. 3). The lack of fumarase in DPANN-HV MAGs suggests they do not utilize fumarate, even if fumarate is generated by phenylphosphate carboxylase and associated enzymes. This suggests new aromatic hydrocarbon utilization pathways are present in this group, beyond the use of phenylphosphate carboxylase.

**FIG 5:**
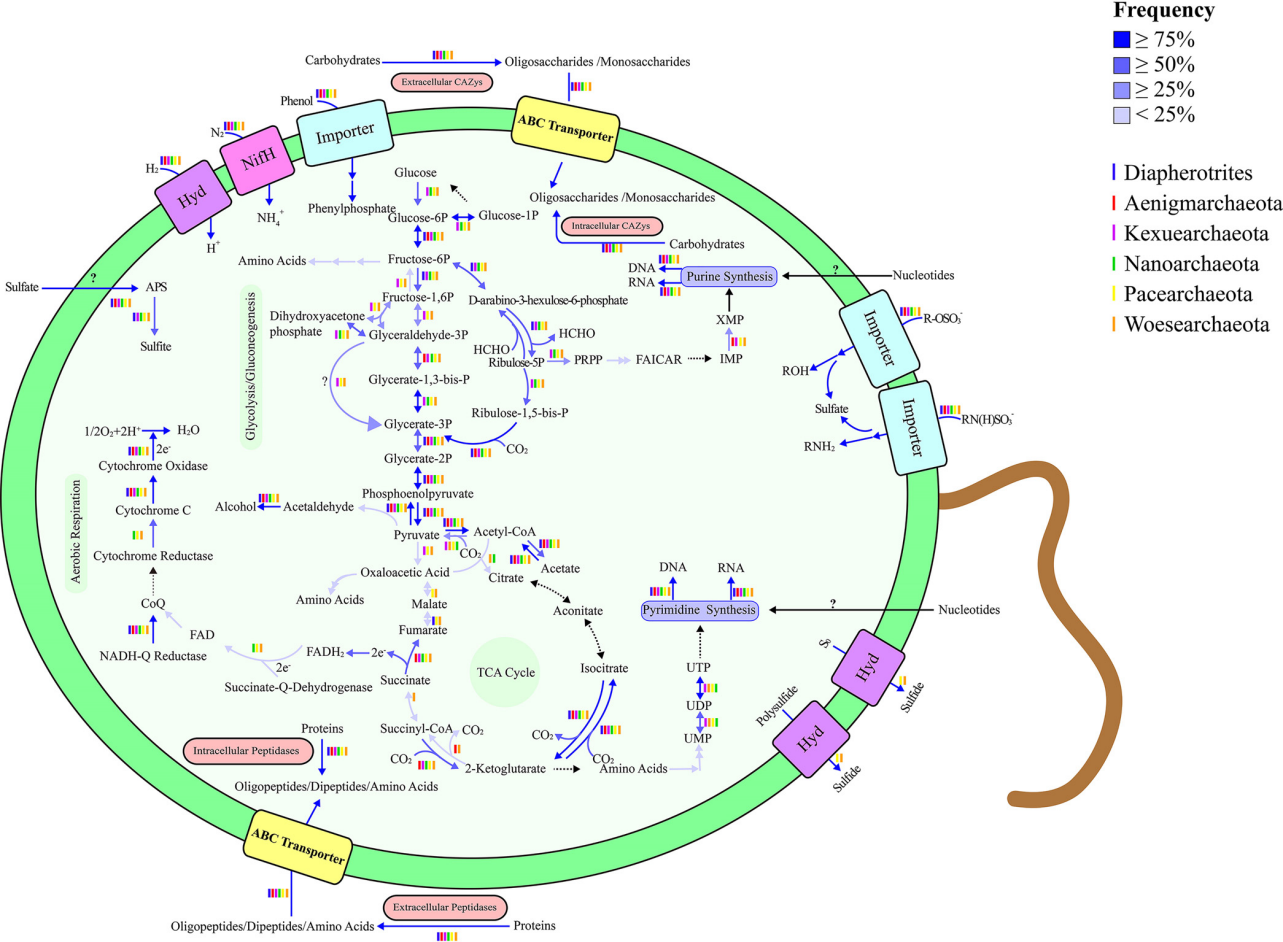
Inferred physiologic capabilities of DPANN-HV. Metabolic pathway predictions were generated using the previous KEGG annotations and core gene analyses. Dashed lines indicate pathways that are absent, and lines with different colors indicate the frequency of pathways present in DPANN-HV genomes. Gene details are provided in Data Set S5. Hyd, hydrogenases; APS, adenosine 5′-phosphosulfate; FAICAR, 5-aminoimidazole-4-carboxamide ribonucleotide; PRPP, phosphoribosyl pyrophosphate.

Considering the harsh nature of deep-sea environments, we next determined whether DPANN-HV can fix carbon dioxide as a means to synthesize organic matter for cell growth. To determine carbon fixation potential, we built a database of genes containing the core enzymes of the Calvin-Benson-Bassham (CBB) cycle, the reductive citric acid (rTCA) cycle, the Wood-Ljungdahl (WL) pathway, the 3-hydroxypropionate-4-hydroxybutyrate cycle, and the dicarboxylate-4-hydroxybutyrate cycle (35). Our results suggest DPANN-HV contain genes encoding ribulose-1,5-bisphosphate carboxylase (a carbon dioxide-fixing enzyme in the CBB cycle), phosphoribulokinase (a core enzyme in the CBB cycle), pyruvate ferredoxin oxidoreductase, 2-oxoglutarate:ferredoxin oxidoreductase, and isocitrate dehydrogenase (carbon dioxide fixing enzymes in the rTCA cycle) (Fig. 3 and 5, Data Set S5).

However, whether DPANN-HV could fix carbon dioxide needs further investigation. On the one hand, pyruvate ferredoxin oxidoreductase, 2-oxoglutarate:ferredoxin oxidoreductase and isocitrate dehydrogenase (carbon dioxide fixing enzymes in the rTCA cycle) are often found in organisms that do not fix carbon, such as *Pyrococcus* (28, 36). On the other hand, both of these pathways are incomplete despite the presence of individual carbon fixation genes. The pentose phosphate pathway, which is important for the CBB cycle, is also absent from DPANN-HV MAGs (37) (Fig. S14). ATP-citrate lyase, citryl-coenzyme A (CoA) lyase, and citryl-CoA synthetase are found in a few MAGs and are also important in the rTCA cycle (Fig. 3) (38, 39). Therefore, the occurrence of carbon fixation in DPANN-HV is still unclear.

### DPANN-HV assimilate nitrogen but are limited in *de novo* nucleotide and amino acid biosynthesis.

As nitrogen is an important element for all living beings, we next investigated the nitrogen cycle in DPANN-HV. Our results show genes encoding nitrogenases are distributed throughout DPANN-HV MAGs (Fig. 3). In addition, some genes encoding nitrogen fixation system regulators were also present in most MAGs, suggesting DPANN-HV can assimilate nitrogen and produce ammonium salts, one of the most important nutrients for microorganisms. Interestingly, DPANN-HV likely lack the ability of *de novo* purine biosynthesis (Fig. S15, Data Set S5). We also found a similar lack of *de novo* pyrimidine biosynthesis (Fig. S16). Therefore, we presume DPANN-HV possesses an alternative metabolism to obtain nucleosides, nucleoside monophosphates, diphosphates, and even triphosphates from their surrounding environment and subsequently process these into DNA and RNA. For example, type IV secretory pathways are present in almost all DPANN-HV MAGs (Data Set S6), indicating DPANN-HV can obtain nucleotides through conjugation (40). Thus, we can presume DPANN-HV prefer environments with relatively high biomass (41), as they need to acquire biological substances from other organisms in their environment. We also found the DPANN-HV seem incapable of synthesizing many amino acids, such as isoleucine and histidine, suggesting that these organisms have limited capacity in amino acid biosynthesis (Fig. 5, Data Set S6). DPANN-HV might obtain amino acids by degrading extracellular proteins with secreted peptidases, whose genes are widespread in their genomes. Alternatively, DPANN-HV might acquire substances they are unable to produce from host cells, as several studies have suggested (13–16). DPANN-HV might obtain nucleotides and amino acids from the environment instead of *de novo* synthesis. This lifestyle is logically suitable for tiny microorganisms, because they are not necessary to carry genes of complete synthesis pathways and consume less energy to obtain necessary substances for life.

### DPANN-HV metabolize hydrogen and sulfur compounds that are abundant in hydrothermal ecosystems.

Previous reports suggest methane, hydrogen, and sulfur-containing compounds are abundant in hydrothermal vent ecosystems (42). From this, we searched in DPANN-HV MAGs for pathways related to the metabolism of these substances. To determine whether methane metabolism exists in DPANN-HV, we aligned MAGs to methane monooxygenase (reacting under aerobic conditions) and methyl coenzyme M reductase (reacting under anaerobic conditions) sequence databases. Interestingly, no methane metabolism genes were identified in DPANN-HV MAGs, suggesting DPANN-HV are not methanotrophic microbes (Fig. 3). To explore whether DPANN-HV metabolize hydrogen, genes encoding hydrogenases were aligned and analyzed in DPANN-HV MAGs. After alignment and classification, only [FeFe] and [NiFe] family hydrogenases were found, but [Fe] family hydrogenases were absent (Fig. 3). [Fe] family hydrogenases are regarded as enzymes involved in methane metabolism (43, 44), which supports our hypothesis that this superphylum cannot utilize methane. In terms of oxygen tolerance, both oxygen-labile hydrogenases and oxygen-tolerant hydrogenases are found in DPANN-HV MAGs (Fig. S17, Data Set S6), suggesting DPANN-HV are facultative anaerobes. It is worth noting that oxygen-labile hydrogenases function as electron donors and, during fermentation, react with formate, acetate, and alcohol under anaerobic conditions, while oxygen-tolerant hydrogenases can metabolize polysulfides and convert elemental sulfur or polysulfides into sulfide under aerobic conditions. This strongly suggests DPANN-HV contribute to the sulfur cycle of hydrothermal vents ecosystem (45) (Table S3).

To investigate a potential role for DPANN-HV in the sulfur cycle of hydrothermal vent systems, we searched within DPANN-HV MAGs for genes involved in the metabolism of inorganic and organic sulfur compounds. We found relatively few MAGs contained sulfide oxidase genes, despite sulfide being widely abundant in hydrothermal vent environments (Fig. 3). However, the sequences of enzymes capable of catabolizing inorganic sulfur compounds are prevalent in DPANN-HV genomes. For example, genes encoding sulfate adenylyltransferases, which convert sulfate, ATP, and H^+^ into adenosine 5′-phosphosulfate (APS), are present in all DPANN-HV MAGs (46). APS is then dissimilated to sulfite through adenylylsulfate reductase, releasing AMP, H^+^, and an oxidative electron receptor (47). Within DPANN-HV MAGs, we also found *atsA* genes encoding arylsulfatase (48) are broadly distributed, suggesting DPANN-HV metabolize organic sulfur compounds to obtain organic molecules and sulfate for subsequent synthesis of cofactors and amino acids (49). Notably, marine polysaccharides, which are considered the most complex organic molecules in the ocean, are highly sulfonated compared to land polysaccharides (50). These polysaccharides are rich within deep-sea hydrothermal systems due to the high biodiversity of the system (51). Therefore, we propose DPANN-HV decompose marine polysaccharides and drive sulfur and carbon cycles within deep-sea hydrothermal vent systems.

## MATERIALS AND METHODS

### Sampling and storage.

Sediment samples used in this study were collected by *RV KEXUE* from the Okinawa Trough in the Western Pacific (124°22'22.794''E, 25°15'48.582''N, depth of approximately 2,190.86 m; and 126°53'85.659''E, 27°47'21.319''N, depth of approximately 961.24 m) in 2018 and stored at −80°C.

### Metagenomic sequencing, assembly, and binning.

Total DNA from 20-g sediments of each sample was extracted using the Tianen bacterial genomic DNA extraction kit by following the manufacturer’s protocol. Extracts were treated with DNase-free RNase to eliminate RNA contamination. DNA concentration was measured using a Qubit 3.0 fluorimeter. DNA integrity was evaluated by gel electrophoresis, and 0.5 μg of each sample was used to prepare libraries. DNA was sheared into fragments between 50 and ∼800 bp using a Covaris E220 ultrasonicator (Covaris, Brighton, UK). DNA fragments between 150 and ∼250 bp were selected using AMPure XP beads (Agencourt, Beverly, MA, USA) and then were repaired using T4 DNA polymerase (ENZYMATICS, Beverly, MA, USA). These DNA fragments were ligated at both ends to T-tailed adapters and amplified for eight cycles. Finally, amplification products were subjected to a single-strand circular DNA library.

All NGS libraries were sequenced on the BGISEQ-500 platform (BGI, Qingdao, China) to obtain 100-bp paired-end raw reads. Quality control was performed by SOAPnuke (v1.5.6) (setting: -l 20 -q 0.2 -n 0.05 -Q 2 -d -c 0–5 0–7 1) (52). The clean data were assembled using MEGAHIT (v1.1.3) (setting: -min-count 2 –k-min 33 –k-max 83 –k-step 10) (53). Thereafter, metaBAT2 (54), Maxbin2 (55), and Concoct (56) were used to automatically bin from assemblies. Finally, MetaWRAP (57) was used to purify and arrange data into final bins. Completeness and contamination were calculated by CheckM (v1.0.18) (58).

### Annotation.

Gene prediction for individual genomes was performed using Glimmer (v 3.02) (59). Sequences were deduplicated using CD-hit (v 4.6.6) (setting: -c 0.95 -aS 0.9 -M 0 -d 0 -g 1) (60). Genomes were annotated by searching predicted genes against KEGG (Release 87.0) (61), NR (20180814), Swissprot (release-2017_07), and EggNOG (2015-10_4.5v) using Diamond (v0.8.23) by default, and the best hits were chosen. To search for specific metabolic genes, sequence files from several databases, including CAZy (62), MEROPS (63), AnHyDeg (64), and HydDB (65), were used to identify carbohydrate active enzymes, peptidases, anaerobic hydrocarbon degradation genes, and hydrogenases. These files were then used to build databases and aligned to MAGs using Diamond (v0.9.29) with an E value of 1e−5 in sensitive mode. Sequences from NCBI (only archaeal and bacterial nonredundant sequences were selected) and UniProt (only reviewed sequences were selected) were used for further metabolic analyses. An alignment database was generated using Diamond with an E value of 1e−5. Protein localization was determined for CAZymes and peptidases using SignalP (66) and phobius (67, 68) with default parameters. Figures were generated using R (v3.5.1).

### Phylogenetic analysis.

The phylum composition of MAGs within the archaeal domain was determined by Aspera (v3.9.8), using three downloaded NCBI referenced genome sequences per archaeal phylum. Phylosift (v1.0.1) (69) was used to extract 37 marker genes (see Table S2 in the supplemental material) within the genomes with automated settings. Sequences were trimmed using TrimAl (version 1.2) (70) with gappyout function. A maximum likelihood tree was inferred using IQ-TREE (v1.6.12) (71, 72) with the GTR+F+R10 model (-bb 1000) and displayed using iTOL (v5) (73).

DPANN-HV MAG phyla were confirmed by comparison to known DPANN MAGs in the NCBI database. The same methods as those described above were used for the phylogenetic analyses of DPANN MAGs and DPANN-HV genomes, with the exception of using a GTR+F+I+G4 model when using IQ-TREE.

CompareM (v 0.0.23) with aai_wf function (74) was used to calculate the average amino acid identity across all MAGs and NCBI DPANN referenced genomes. Results were displayed as a heatmap using R (3.5.1).

### Data availability.

All referenced genomes were retrieved from the NCBI RefSeq database. All core genes were obtained from the NCBI nonredundant protein database (filter: the sequences only belonging to bacteria and archaea), the UniProt reviewed database, and CAZY, MEROPS, HydDB, and AnHyDeg databases. Additional data supporting the manuscript are included in Data Sets S1 to S6. All assembled sequence data and sample information are available at NCBI under BioProject no. PRJNA593679.

## Supplementary Material

Supplemental file 6

Supplemental file 7

Supplemental file 3

Supplemental file 2

Supplemental file 5

Supplemental file 1

Supplemental file 4
