# Supplementary material for: Metagenomic Insights into the Metabolic and Ecological Functions of Abundant Deep-Sea Hydrothermal Vent DPANN Archaea
Source: Appl Environ Microbiol. 2021 Apr 13;87(9):e03009-20. doi: 10.1128/AEM.03009-20 (PMC8091004; doi:10.1128/AEM.03009-20)
Supplement: Supplemental file 1 [file AEM.03009-20-s0001.pdf]

Supplementary information

Metagenomic insights into the metabolic and ecological functions of abundant deep-sea hydrothermal vent DPANN archaea

Ruining Cai[1,2,3,4], Jing Zhang[1,2,3,4], Rui Liu[1,2,4], Chaomin Sun[1,2,4*]

[1]CAS Key Laboratory of Experimental Marine Biology & Center of Deep Sea Research, Institute of Oceanology, Chinese Academy of Sciences, Qingdao, China

[2]Laboratory for Marine Biology and Biotechnology, Pilot National Laboratory for Marine Science and Technology, Qingdao, China

[3]College of earth science, University of Chinese Academy of Sciences, Beijing, China

[4]Center of Ocean Mega-Science, Chinese Academy of Sciences, Qingdao, China

[*] Corresponding author

Chaomin Sun Tel.: +86 532 82898857; fax: +86 532 82898857.

E-mail address: sunchaomin@qdio.ac.cn

Table S1. The geographic positions of sampling sites and nearby hydrothermal vents.

Type	Name	Longitude (E, °)	Latitude (N, °)
Nearby hydrothermal vent	Leyte	125.00	11.00
Nearby hydrothermal vent	Philippine Ridge; West Philippine Basin	131.42	12.00
Nearby hydrothermal vent	Batan	122.00	20.00
Nearby hydrothermal vent	Taketomi Thermal Area	124.10	24.35
Nearby hydrothermal vent	Kueishan Island;	122.00	24.83
Nearby hydrothermal vent	Yonaguni Knoll IV	122.70	24.85
Nearby hydrothermal vent	SPOT; Hatoma Knoll	123.84	24.86
Nearby hydrothermal vent	SPOT; SPOT-5	122.70	24.87
Nearby hydrothermal vent	Yaeyama Graben	124.88	25.23
Nearby hydrothermal vent	Izena Cauldron	127.08	27.27
Nearby hydrothermal vent	Iheya Ridge	126.98	27.54
Nearby hydrothermal vent	Natsushima 84-1 Knoll	127.14	27.58
Nearby hydrothermal vent	North Knoll; Iheya Ridge	126.90	27.79
Nearby hydrothermal vent	Minami-Ensei Knoll	127.64	28.39
Nearby hydrothermal vent	Okinawa Trough; Site ES2	128.10	28.81
Nearby hydrothermal vent	Okinawa Trough; Site ES1	129.08	30.33
Nearby hydrothermal vent	Nagahama Bay; Iwodake Volcano	130.33	30.75
Nearby hydrothermal vent	Kagoshima Bay; Sakura-jima Volcano	130.80	31.65
Sampling site	north-191	126.54	27.47
Sampling site	south	124.22	25.15

Table S2. Marker genes used in phylogenetic analysis.

ID	Protein
DNGNGWU00001	ribosomal protein S2 rpsB
DNGNGWU00002	ribosomal protein S10 rpsJ
DNGNGWU00003	ribosomal protein L1 rplA
DNGNGWU00005	translation initiation factor IF-2
DNGNGWU00006	metalloendopeptidase
DNGNGWU00007	ribosomal protein L22
DNGNGWU00009	ribosomal protein L4/L1e rplD
DNGNGWU00010	ribosomal protein L2 rplB
DNGNGWU00011	ribosomal protein S9 rpsI
DNGNGWU00012	ribosomal protein L3 rplC
DNGNGWU00013	phenylalanyl-tRNA synthetase beta subunit
DNGNGWU00014	ribosomal protein L14b/L23e rplN
DNGNGWU00015	ribosomal protein S5
DNGNGWU00016	ribosomal protein S19 rpsS
DNGNGWU00017	ribosomal protein S7
DNGNGWU00018	ribosomal protein L16/L10E rplP
DNGNGWU00019	ribosomal protein S13 rpsM
DNGNGWU00020	phenylalanyl-tRNA synthetase alpha subunit
DNGNGWU00021	ribosomal protein L15
DNGNGWU00022	ribosomal protein L25/L23
DNGNGWU00023	ribosomal protein L6 rplF
DNGNGWU00024	ribosomal protein L11 rplK
DNGNGWU00025	ribosomal protein L5 rplE
DNGNGWU00026	ribosomal protein S12/S23
DNGNGWU00027	ribosomal protein L29
DNGNGWU00028	ribosomal protein S3 rpsC
DNGNGWU00029	ribosomal protein S11 rpsK
DNGNGWU00030	ribosomal protein L10
DNGNGWU00031	ribosomal protein S8
DNGNGWU00032	tRNA pseudouridine synthase B
DNGNGWU00033	ribosomal protein L18P/L5E
DNGNGWU00034	ribosomal protein S15P/S13e
DNGNGWU00035	Porphobilinogen deaminase
DNGNGWU00036	ribosomal protein S17
DNGNGWU00037	ribosomal protein L13 rplM
DNGNGWU00039	ribonuclease HII
DNGNGWU00040	ribosomal protein L24

*The DNGNGWU marker genes in phylosift refer to a suite of single-copy, protein-coding marker genes. All 37 DNGNGWU marker genes were concatenated to construct maximum likelihood phylogenetic tree.

Table S3. The sulfhydrogenase subunit in the group 3b hydrogenases of DPANN-HV

Protein_id	Bin	Protein clusters
WP_029915222.1	H2.bin.12	sulfur reductase subunit alpha
WP_028842337.1	H2.bin.54	sulfur reductase subunit alpha
WP_013905579.1	H1.bin.22	sulfhydrogenase 1 subunit alpha
WP_012970030.1	H2.bin.48	sulfur reductase subunit alpha
WP_012506474.1	H2.bin.78	/
WP_011416040.1	H2.bin.75	sulfur reductase subunit alpha /
WP_008085116.1	H1.bin.21	sulfhydrogenase 1 subunit alpha
WP_008085116.1	H1.bin.39	sulfhydrogenase 1 subunit alpha
CUU06124.1	H2.bin.89	/

Table S4. The classes and representative sequences of fructose-1,6-bisphosphate (FBP) aldolase and phosphatase

Enzymes	Uniprot_ID	Reference
FBP aldolase class I	Q07159	(1)
FBP aldolase class II	AAF22441	(2)
FBP aldolase class IA	BAC21177	(3)
ADH synthase	NP_247374	(4)
FBPase class I	NP_418653	(5)
FBPase class II	P0A9C9	(6)
FBPase class III	Q45597	(7)
FBPase class IV	NP_247073	(8)
FBPase class V<br>Fructose 1,6-bisphosphate aldolase/phosphatase	BAC10571	(3)

*Referring to the following article, these genes were chosen (9).

*Abbreviations: FBP aldolase: fructose-1,6-bisphosphate aldolase; FBPase: fructose-1,6-bisphosphatase;

References:

1. Witke C, Götz F. 1993. Cloning, sequencing, and characterization of the gene encoding the class I fructose-1,6-bisphosphate aldolase of Staphylococcus carnosus. Journal of bacteriology 175:7495-9.
2. De Montigny C, Sygusch J. 1996. Functional characterization of an extreme thermophilic class II fructose-1,6-bisphosphate aldolase. European journal of biochemistry 241:243-248.
3. Rashid N, Imanaka H, Kanai T, Fukui T, Atomi H, Imanaka T. 2002. A novel candidate for the true fructose-1,6-bisphosphatase in archaea. Journal of biological chemistry 277:30649-30655.
4. White RH. 2004. Aspartate semialdehyde and a 6-deoxy-5-ketohexose 1-phosphate are the precursors to the aromatic amino acids in *Methanocaldococcus jannaschii*. Biochemistry 43:7618-7627.
5. Babul J, Guixé V. 1983. Fructose bisphosphatase from *Escherichia coli*. purification and characterization. Archives of biochemistry and biophysics 225:944-949.
6. Donahue JL, Bownas JL, Niehaus WG, Larson TJ. 2000. Purification and characterization of *glp*-encoded fructose 1,6-bisphosphatase, a new enzyme of the glycerol 3-phosphate regulon of *Escherichia coli*. Journal of bacteriology 182:5624-5627.
7. Fujita Y, Freese E. 1979. Purification and properties of fructose-1,6-bisphosphatase of *Bacillus subtilis*. Journal of biological chemistry 254:5340-5349.
8. Stec B, Yang H, Johnson KA, Chen L, Roberts MF. 2000. MJ0109 is an enzyme that is both an inositol monophosphatase and the 'missing' archaeal fructose-1,6-bisphosphatase. Nature structural biology 7:1046-1050.
9. Say RF, Fuchs G. 2010. Fructose 1,6-bisphosphate aldolase/phosphatase may be an ancestral gluconeogenic enzyme. Nature 464:1077-1081.

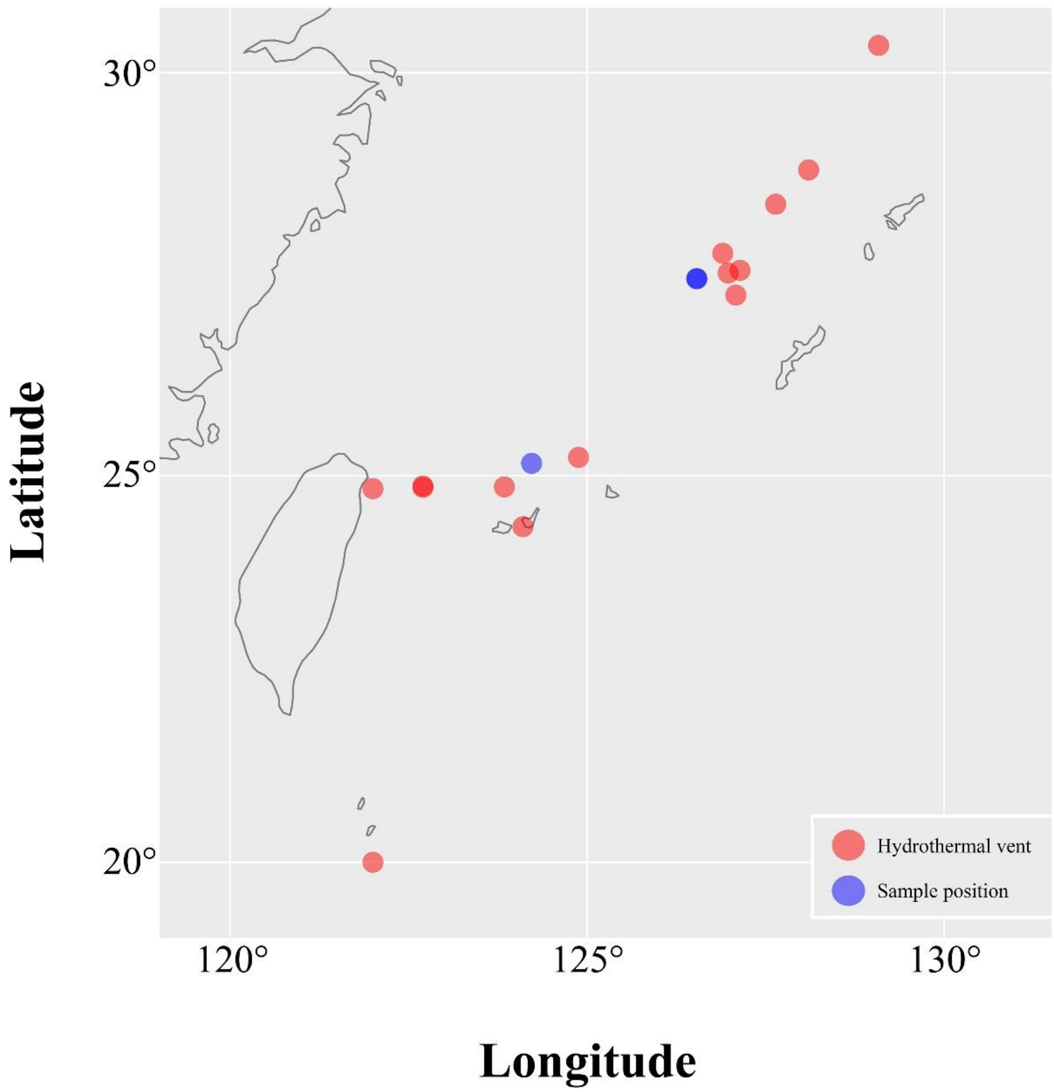

Fig. S1. Overview of sampling sites. The blue points: sampling sites; The red points: nearby hydrothermal vents. Sediments from sampling sites were used for DNA extraction and metagenomics analysis. Metadata for all sites are summarized in Table S1.

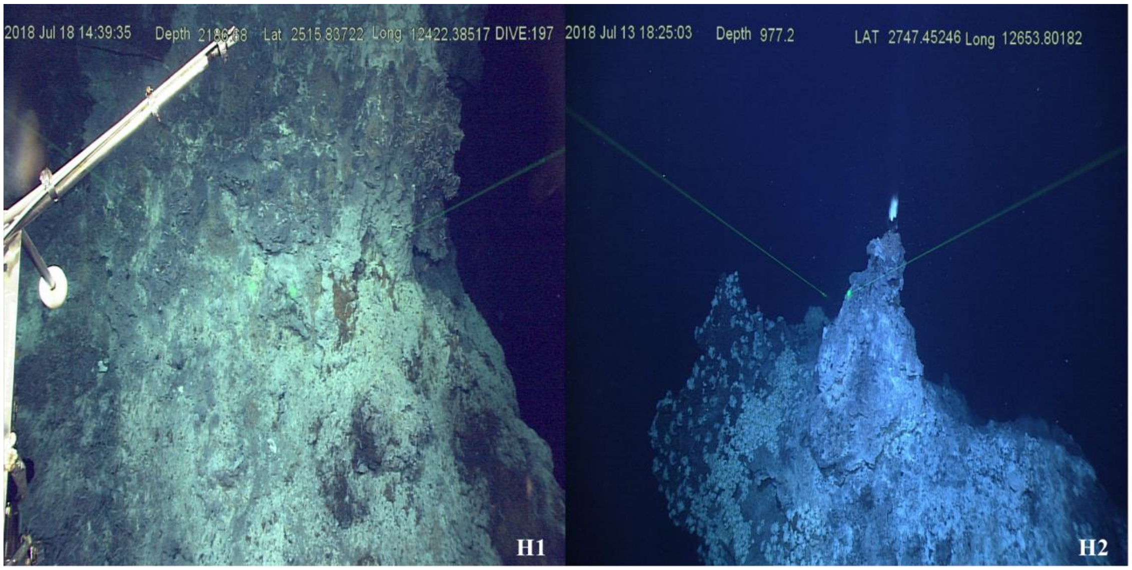

Fig S2. *In situ* photos of sampling sites. In these locations, the typical characteristics of deep-sea hydrothermal areas such as the faint glow from the black smoker and a large number of shrimps were observed.

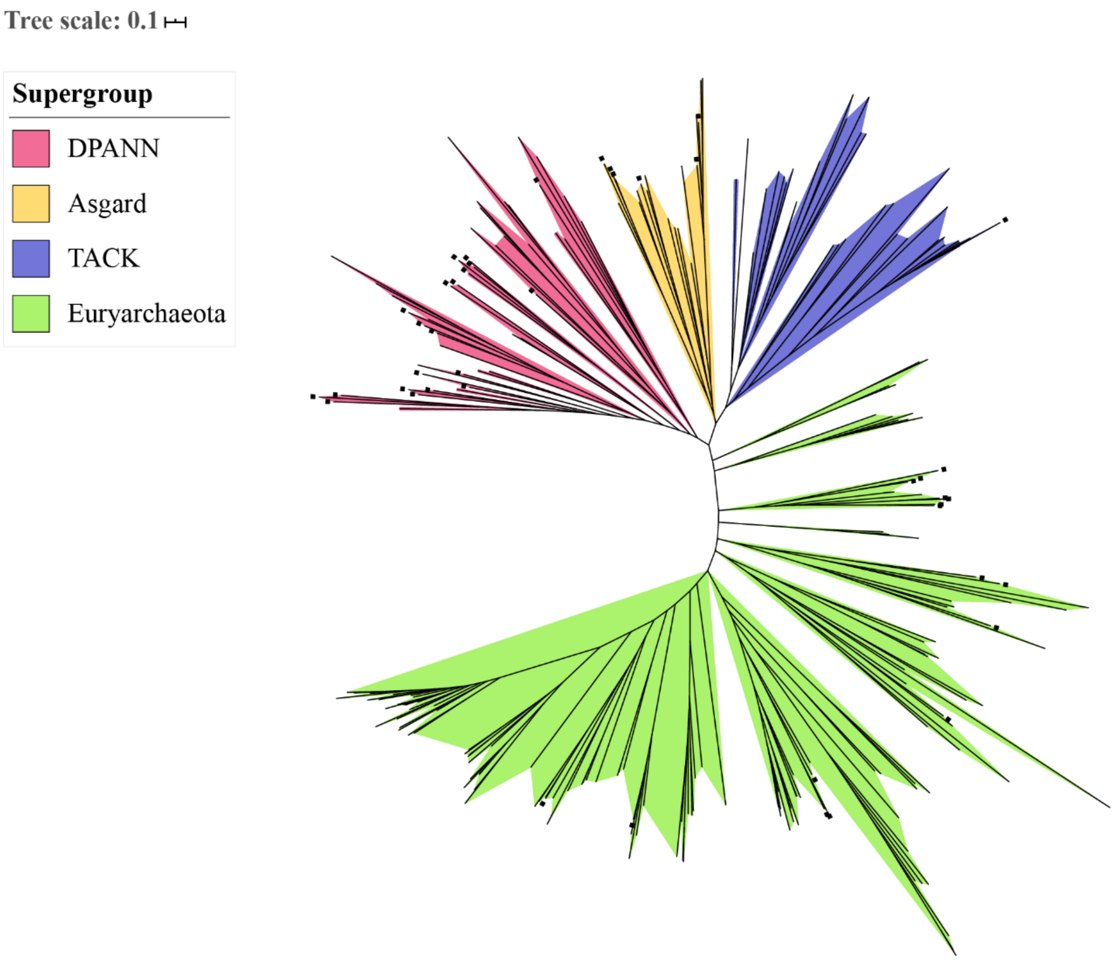

Fig. S3. Maximum likelihood phylogenetic tree of referenced archaeal genomes and the DPANN-HV genomes. The red region: DPANN; the yellow region: Asgard; the blue region: TACK; the green region: Euryarchaeota. We used all archaeal genomes assembled from sediments to do phylogenetic analyses and to investigate their distribution. The referenced genes and genomes are recorded in the Table S2 and DATASET S2.

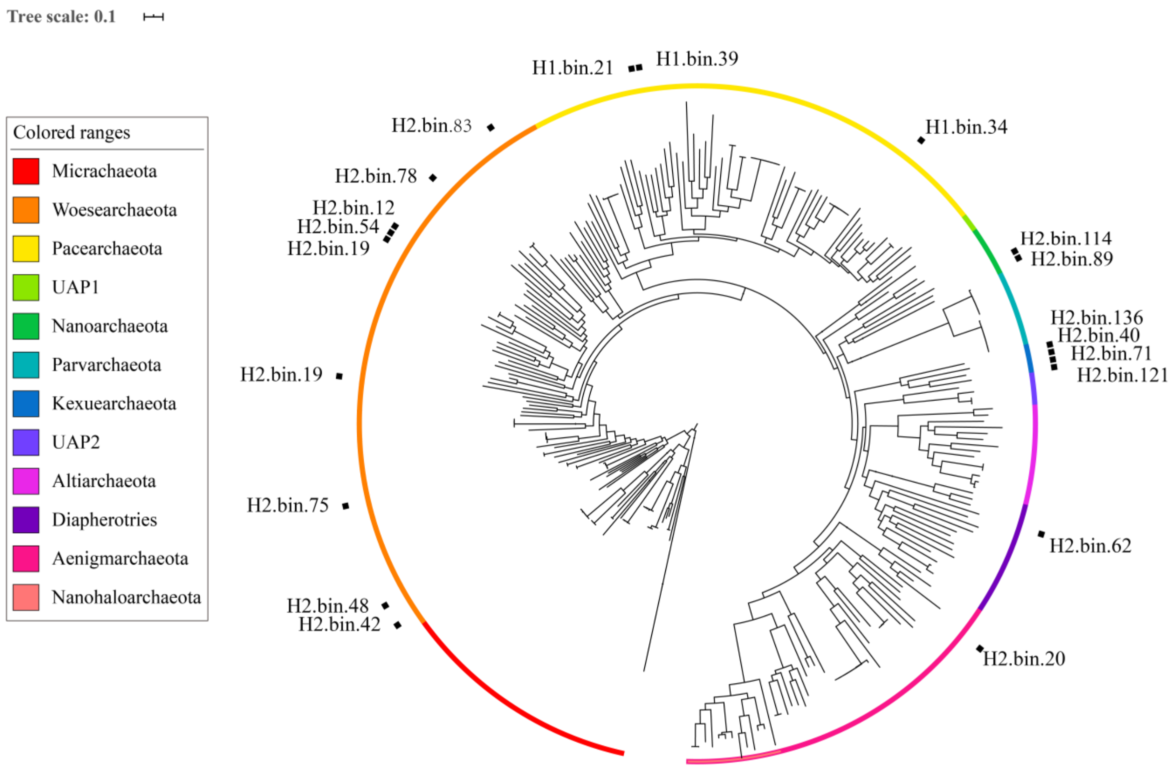

Fig. S4. Maximum likelihood phylogenetic tree of referenced DPANN genomes and the DPANN-HV genomes. Used genomes contain the DPANN genomes collected from NCBI and the DPANN-HV assembled genomes. The tree was used for confirming the phylum of each assembled genome. The referenced genes and genomes are recorded in the Table S2 and DATASET S3.

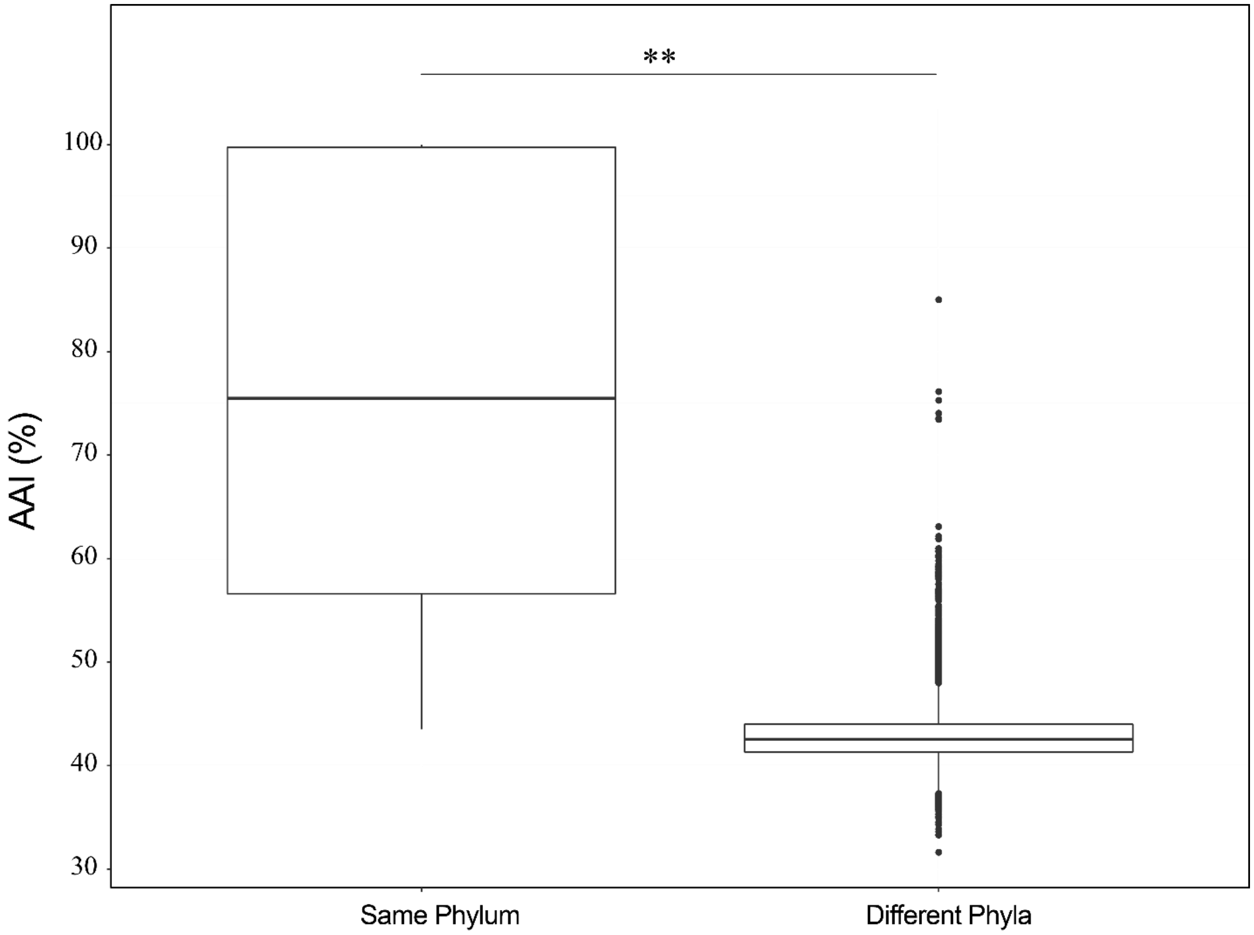

Fig. S5. Average amino acid identity of the same phylum and the different phyla in referenced DPANN genomes. The amino acid identity was calculated by CompareM. All data are available in DATASET S4.

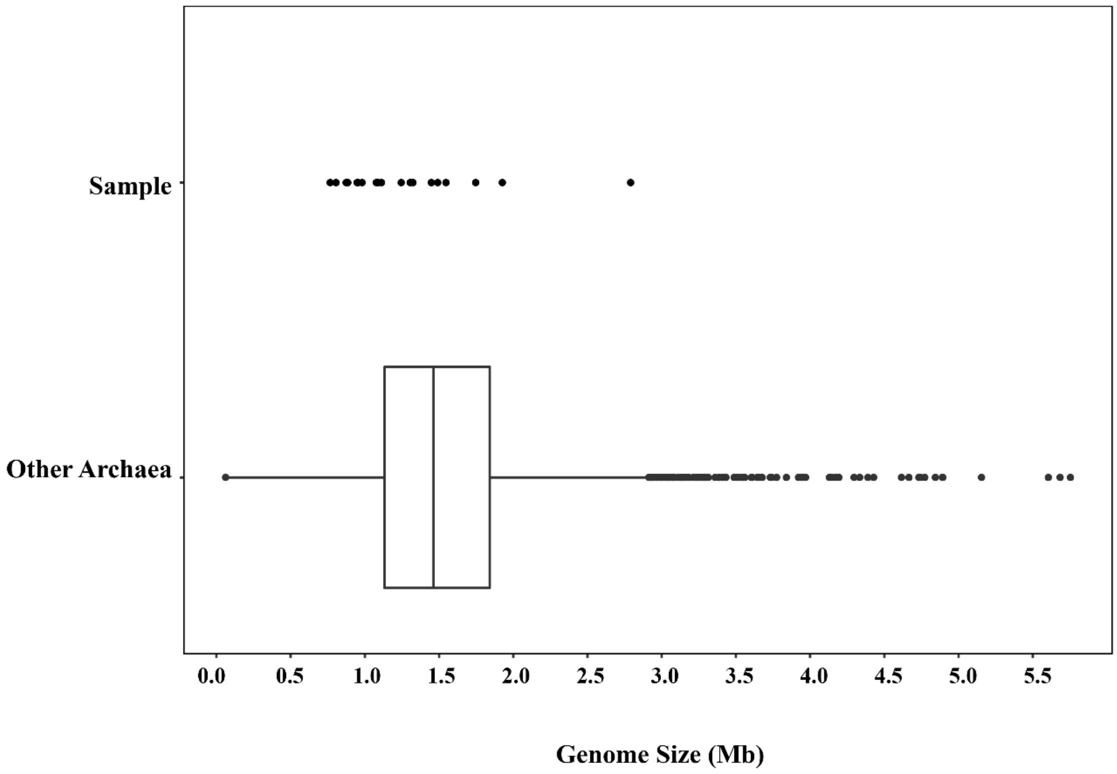

Fig. S6. The size ranges of the DPANN-HV genomes and referenced genomes. The referenced genomes contain all archaeal genome sequences except DPANN's in NCBI referenced genomes. All data are available in DATASET S3.

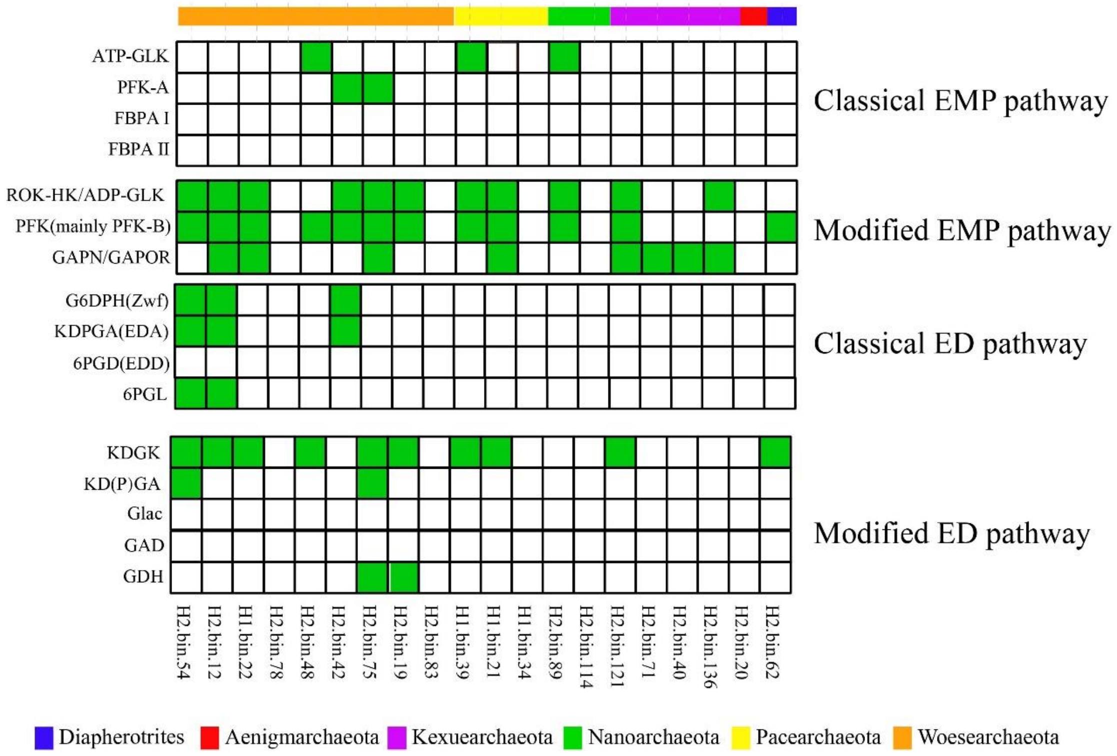

Fig. S7. The distribution of different genes in classical and modified Embden-Meyerhof-Parnas (EMP) pathway as well as Entner-Doudoroff (ED) pathway. ATP-GLK: ATP-dependent glucokinase; PFK-A: ATP-dependent phosphofructokinase (phosphofructokinase family); FBPA I: fructose-1,6-bisphosphate aldolase class I; FBPA II: fructose-1,6-bisphosphate aldolase class II; ROK-HK: ROK hexokinase; PFK: phosphofructokinase; PFK-B: ATP-dependent phosphofructokinase (PFK-B family); ADP-GLK: ADP-dependent glucokinase; GAPN: non-phosphorylating glyceraldehyde-3-phosphate dehydrogenase; GAPOR: ferredoxin (Fd)-dependent glyceraldehyde-3-phosphate oxidoreductase; G6DPH(Zwf): glucose-6-phosphate dehydrogenase (encoded by *Zwf* gene); KDPGA(EDA): 2-keto-3-deoxy-6-phosphogluconate aldolase (Entner-Doudoroff aldolase); 6PGD: gluconate-6-phosphate dehydratase; 6PGL: 6-phosphogluconate-d-lactonase; KDGK: 2-keto-3-deoxy-d-gluconate kinase; KD(P)GA: 2-keto-3-deoxy-(6-phospho)gluconate aldolase; Glac: gluconolactonase; GAD: gluconate dehydratase; GDH: glucose dehydrogenase. ”/” in this picture is used to present enzymes catalyzing the same substrates and generating the same products used in EMP or ED pathways.

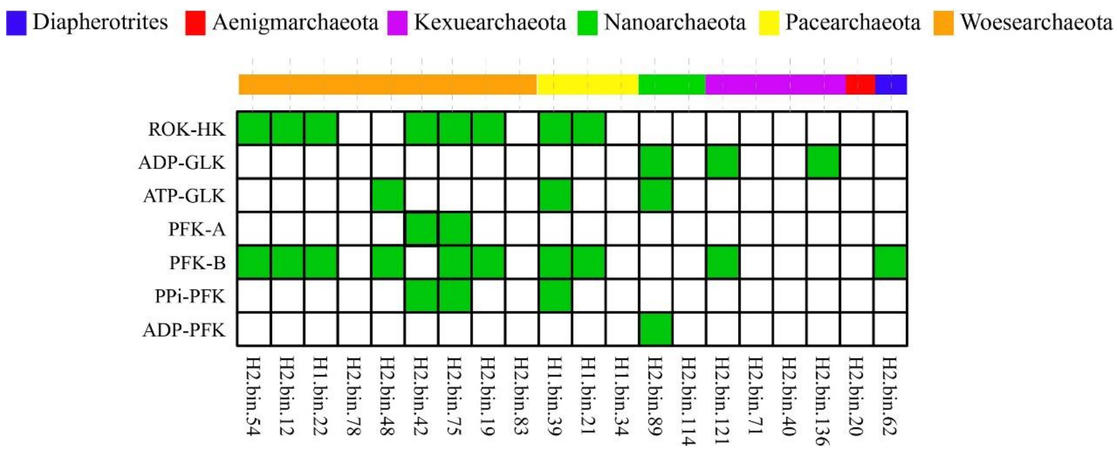

Fig. S8. Different types of glucokinase and phosphofructokinase found in DPANN-HV. ROK-HK: ROK hexokinase; ADP-GLK: ADP-dependent glucokinase; ATP-GLK: ATP-dependent glucokinase; PFK-A: ATP-dependent phosphofructokinase (phosphofructokinase family); PFK-B: ATP-dependent phosphofructokinase (PFK-B family); PP_i-PFK: PP_i-dependent phosphofructokinase; ADP-PFK: ADP-dependent phosphofructokinase;

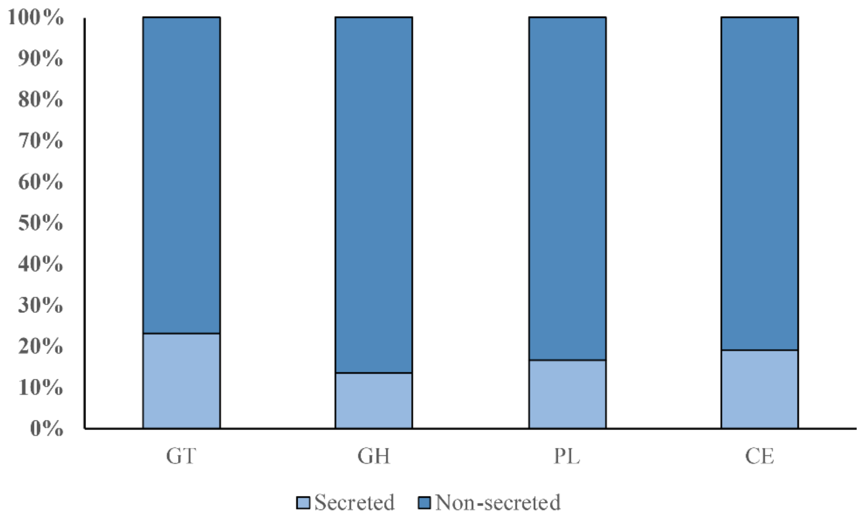

Fig. S9. Relative abundance of genes encoding secreted and non-secreted CAZymes in the DPANN-HV assembled genomes. The secreted CAZymes were analyzed using SignalP and phobius. All data are available in DATASET S6.

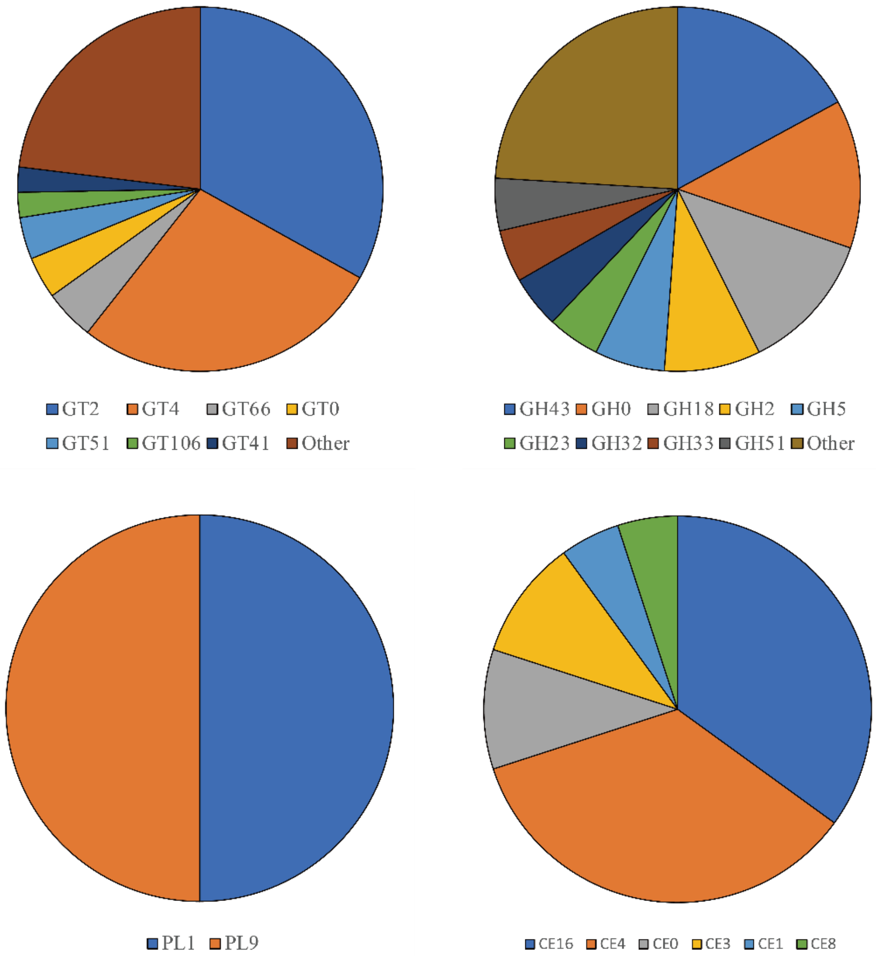

Fig. S10. The composition of the secreted CAZymes in the DPANN-HV genomes. The secreted CAZymes were analyzed using SignalP and phobius. All data are available in DATASET S6.

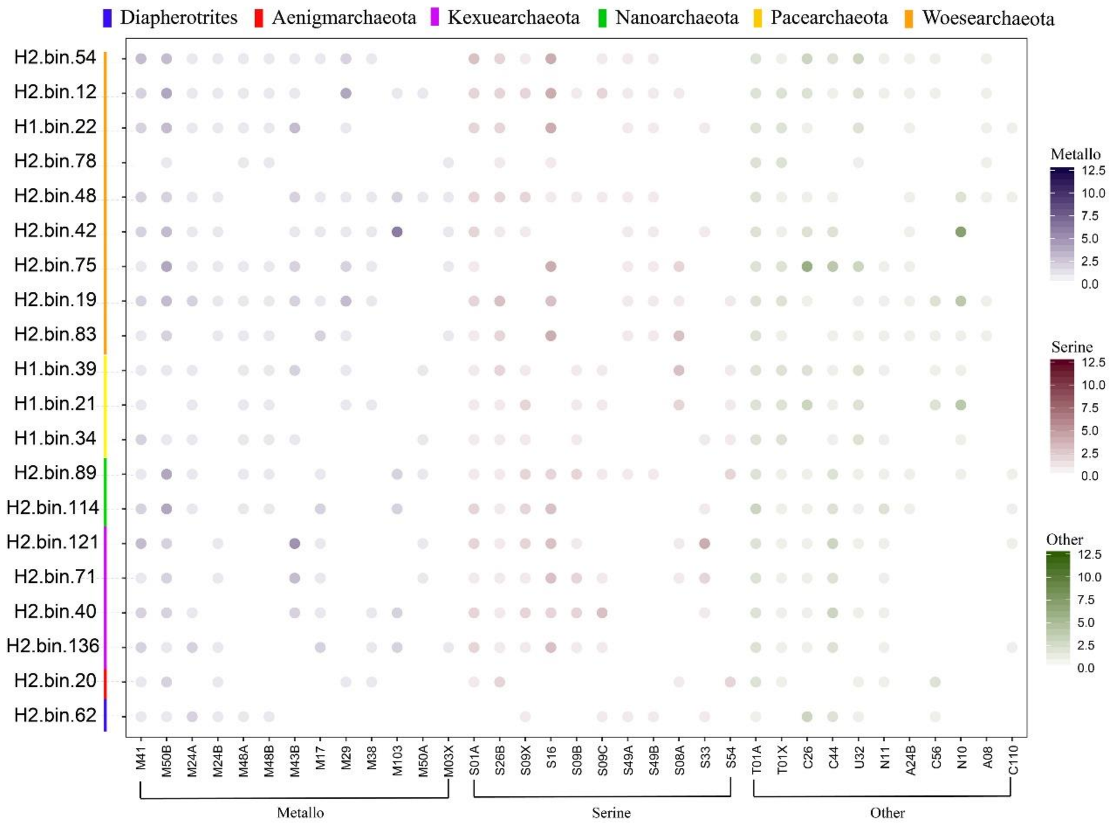

Fig. S11. Relative abundance of genes encoding peptidases in the DPANN-HV assembled genomes. The percentage of genes encoding metallo, serine and other peptidases in each genome was summarized. Numbers of genes belonging to different peptidases families per genome are presented by circles with different colors. All data are available in DATASET S6.

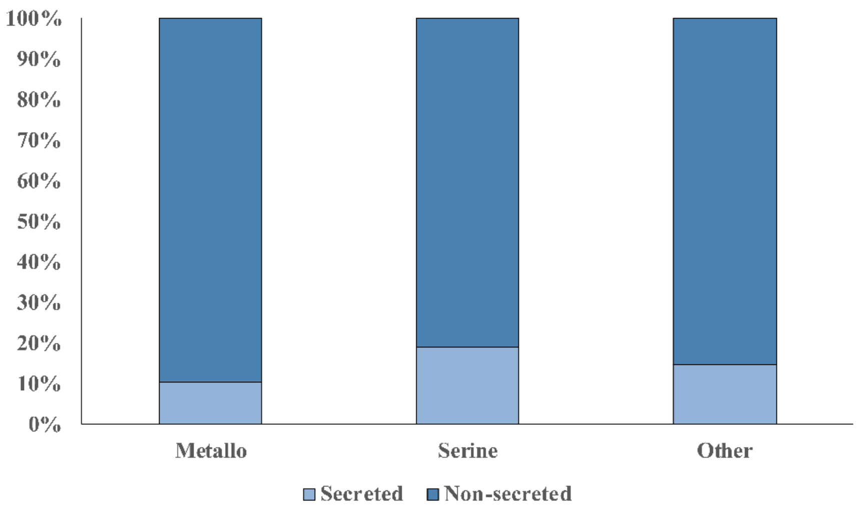

Fig. S12. Relative abundance of genes encoding secreted and non-secreted peptidases in the DPANN-HV assembled genomes. The secreted peptidases were analyzed using SignalP and phobius. All data are available in DATASET S6.

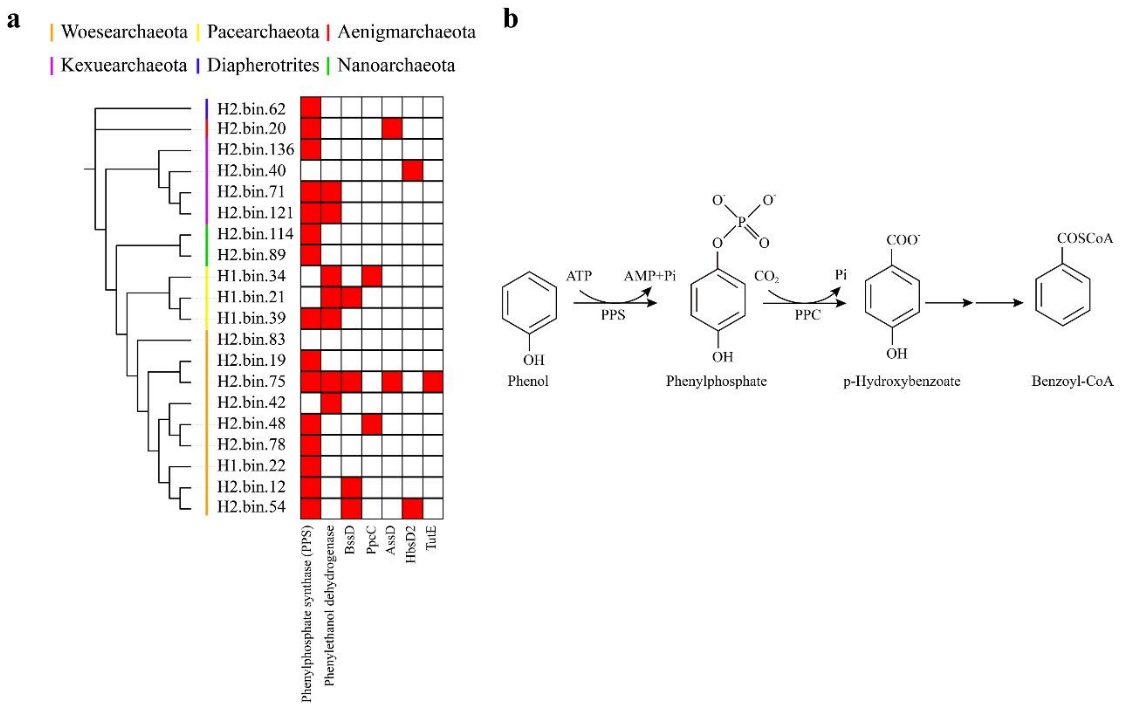

Fig. S13. The distribution of anaerobic hydrocarbon degradation genes and the mainly possible reaction in the DPANN-HV group. (A) The distribution of anaerobic hydrocarbon degradation genes in the DPANN-HV; (B) The mainly possible reactions of anaerobic hydrocarbon degradation in the DPANN-HV.

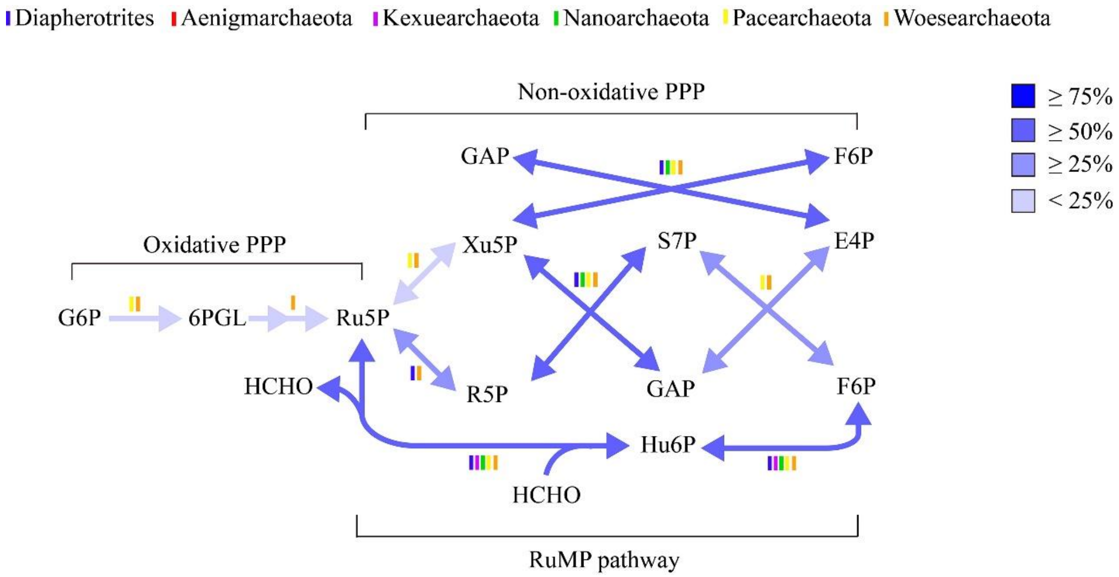

Fig. S14. The oxidative pentose phosphate pathway (PPP), non-oxidative PPP and the ribulose monophosphate (RuMP) pathway in DPANN-HV. G6P, glucose 6-phosphate; 6PGL, 6-phosphoglucono-δ-lactone; Ru5P, ribulose 5-phosphate; Xu5P, xylulose 5-phosphate; R5P, ribose 5-phosphate; S7P, sedoheptulose 7-phosphate; GAP, glyceraldehyde 3-phosphate; E4P, erythrose 4-phosphate; F6P, fructose 6-phosphate; Hu6P, D-arabino-3-hexulose-6-phosphate.

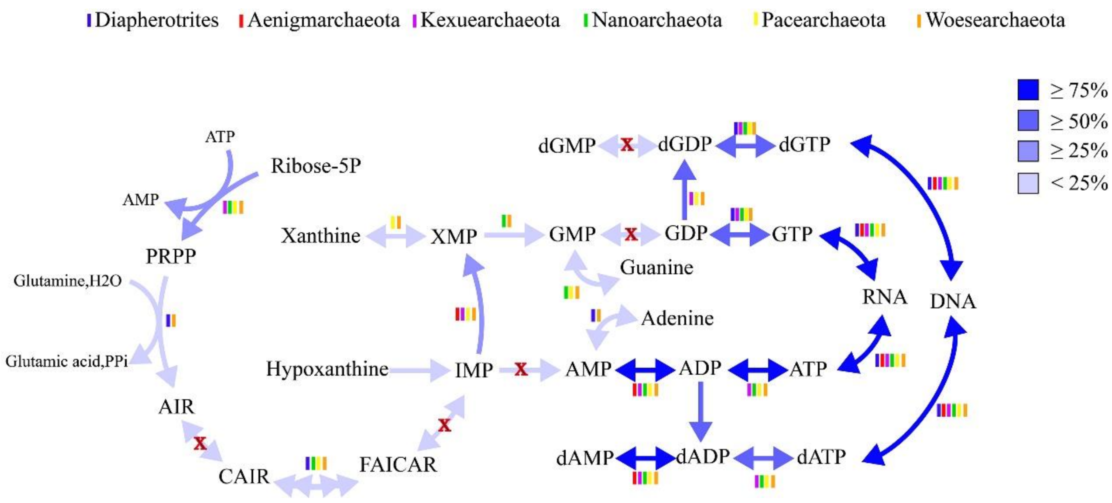

Fig. S15. Purine synthesis pathway in the DPANN-HV. The genes were predicted by KAAS, and the different colors were used for presenting the distribution of genes. PRPP: phosphoribosyl pyrophosphate; AIR: 5-aminoimidazole ribotide; CAIR: 5-phosphoribosyl-4-carboxy-5-aminoimidazole; FAICAR: 5-formamidoimidazole-4-carboxamide ribotide. All data are available in DATASET S5.

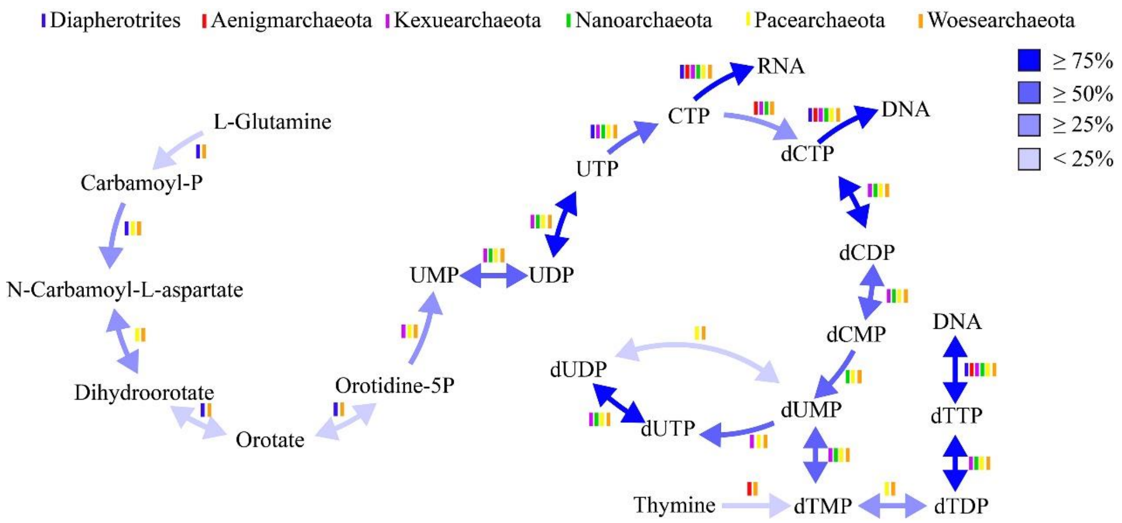

Fig. S16. Pyrimidine synthesis pathway in the DPANN-HV. The genes were predicted by KAAS, and the different colors were used for presenting the distribution of genes. All data are available in DATASET S5.

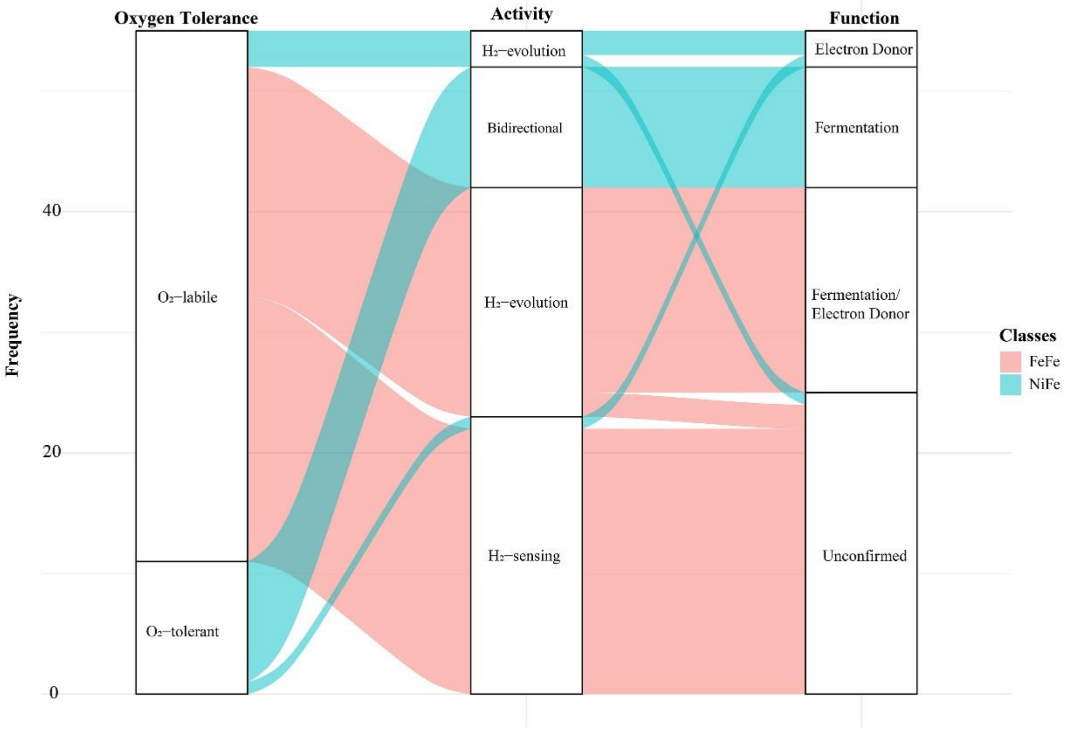

Fig. S17. The oxygen tolerance, activities and functions of hydrogenases encoded in the DPANN-HV genomes. Hydrogenases and features were detected by aligning to the HydDB database. All data are available in DATASET S6.
